# Supplementary figures and images for: Single-Dimensional Human Brain Signals for Two-Dimensional Economic Choice Options
Source: J Neurosci. 2021 Mar 31;41(13):3000–13. doi: 10.1523/JNEUROSCI.1555-20.2020 (PMC8018883; doi:10.1523/JNEUROSCI.1555-20.2020)

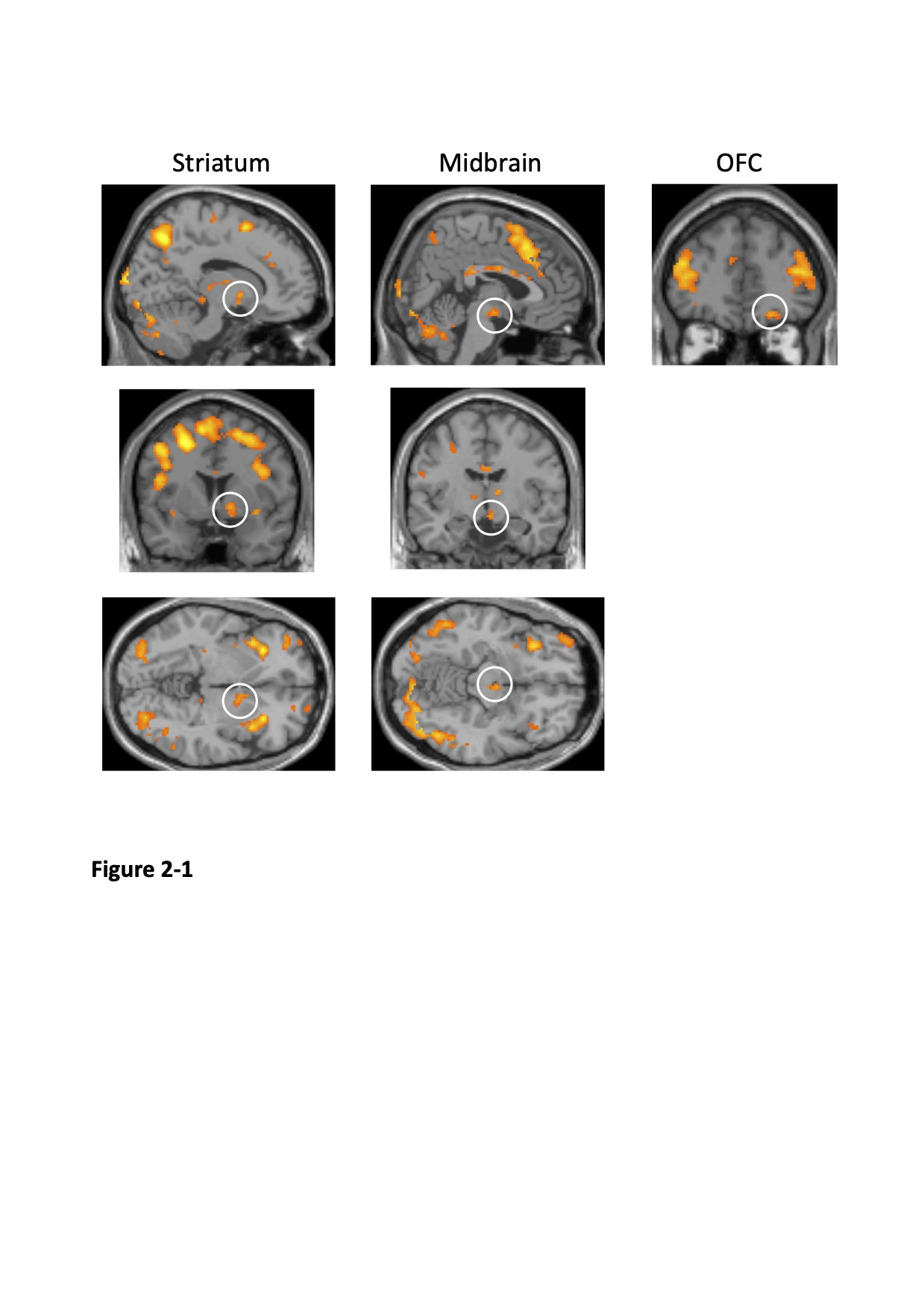

Supplement: Extended Data Figure 2-1 — BOLD responses discriminating bundles between ICs identified with F contrast (map threshold p < 0.005, extent threshold ≥ 10 voxels, high > low), but no discrimination between bundles along same ICs (map threshold p > 0.005; i.e., exclusive mask for brain response to bundles on same ICs with threshold p = 0.005) in a group analysis. Download Figure 2-1, TIF file. [file ns-JN-RM-1555-20-s05.tif]

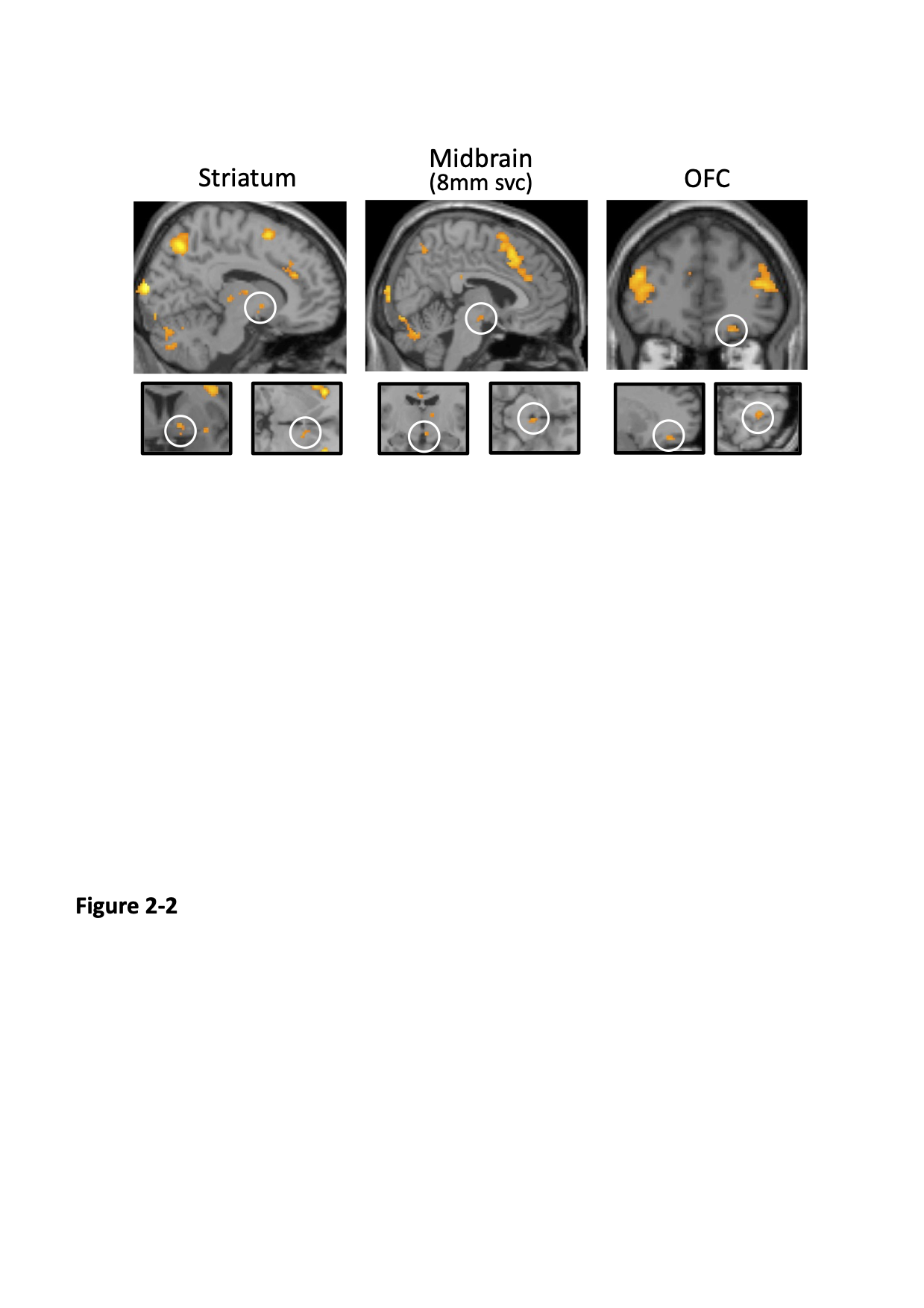

Supplement: Extended Data Figure 2-2 — BOLD responses discriminating bundles between ICs with lower threshold (map threshold p < 0.001, extent threshold ≥ 10 voxels, high > low), but no discrimination between bundles along same ICs with T contrast (map threshold p > 0.005; i.e., exclusive mask for brain response to bundles on same ICs with threshold p = 0.005) in a group analysis. Svc, small volume corrected. Download Figure 2-2, TIF file. [file ns-JN-RM-1555-20-s04.tif]

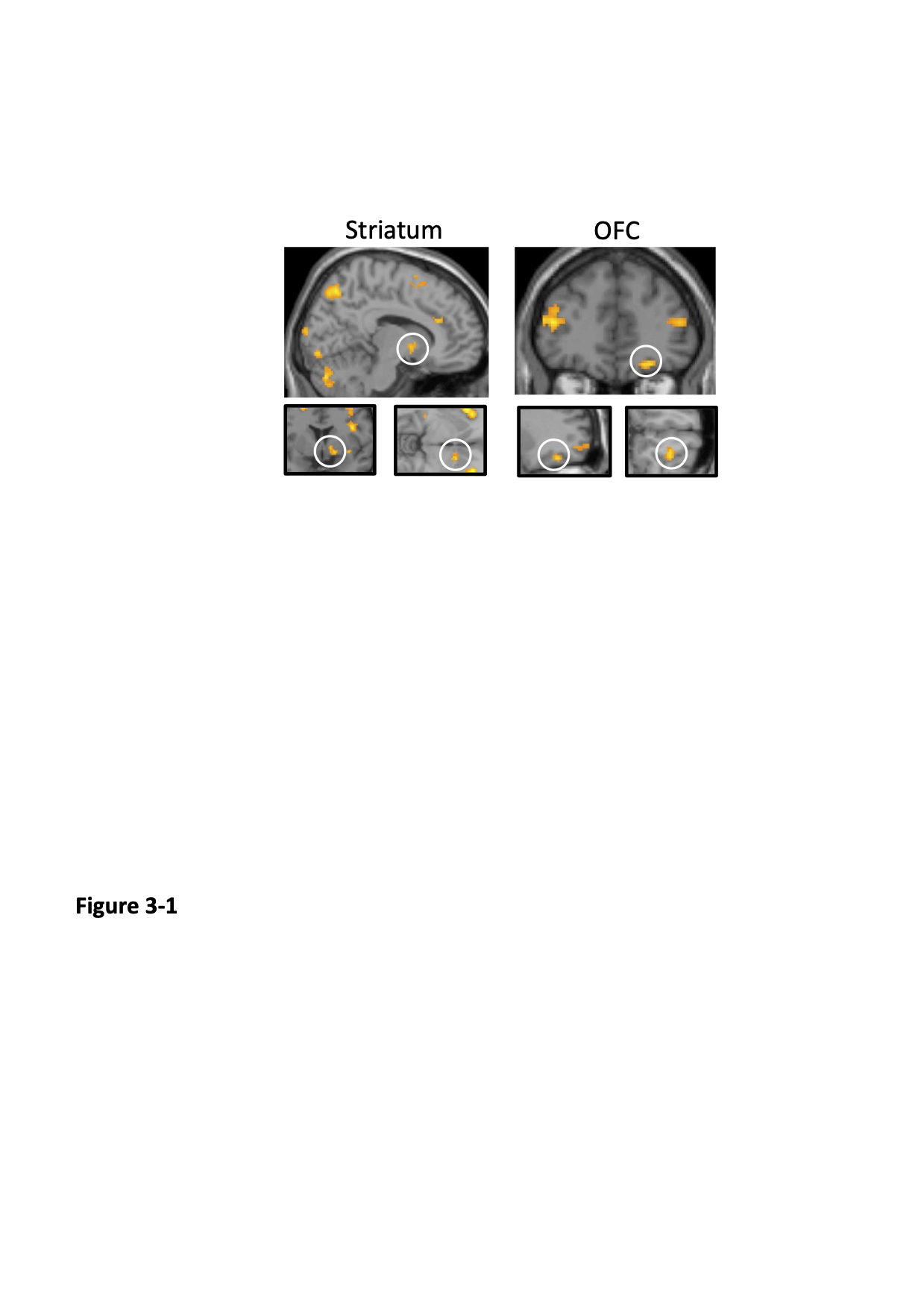

Supplement: Extended Data Figure 3-1 — Higher BOLD responses to more preferred (but physically partially dominated) bundles positioned on different ICs with stricter thresholds (map threshold p < 0.001, extent threshold ≥ 10 voxels) in striatum (left) and OFC (right). Download Figure 3-1, TIF file. [file ns-JN-RM-1555-20-s02.tif]

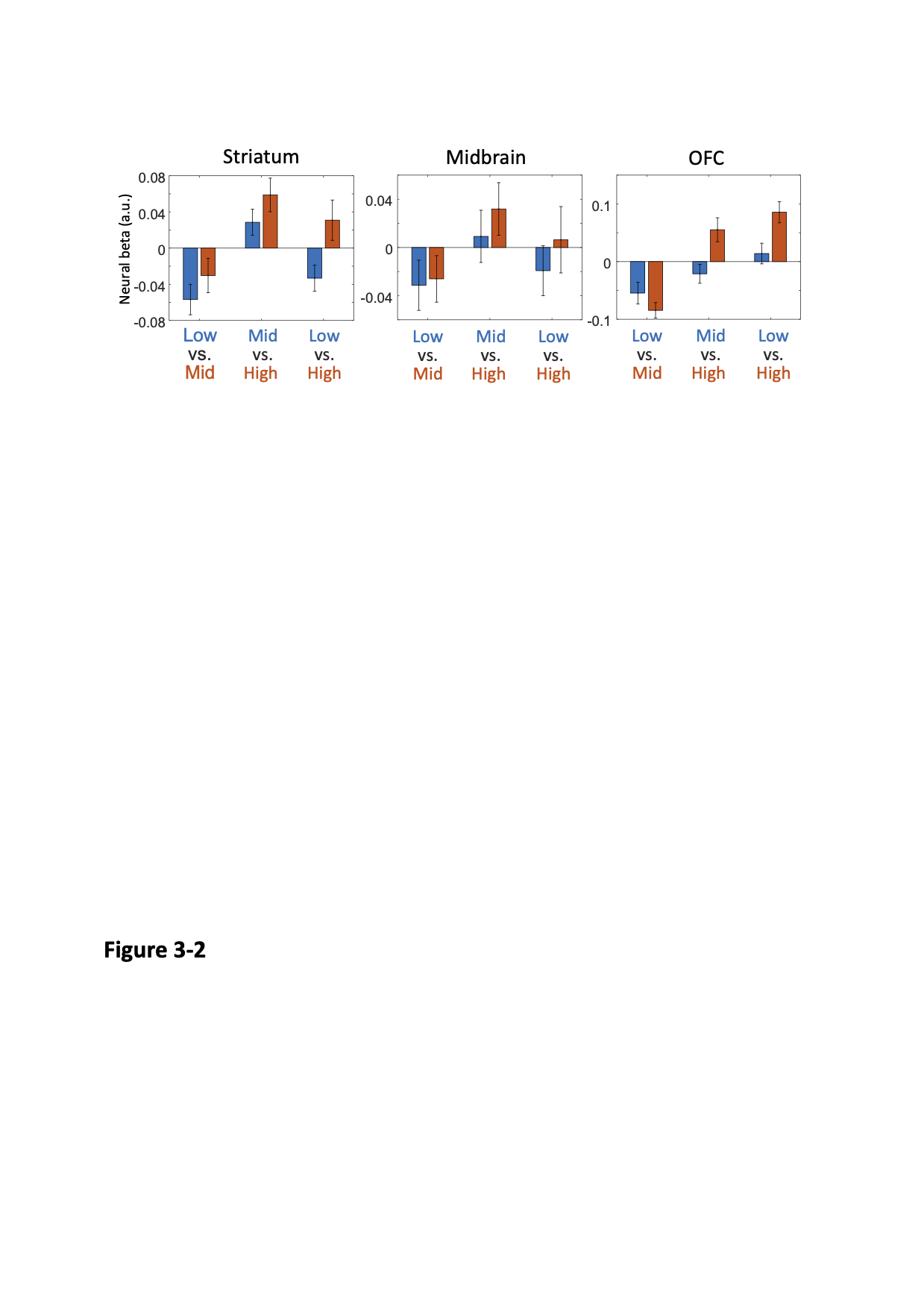

Supplement: Extended Data Figure 3-2 — Bar charts showing neural β coefficients of regression at peak voxels in ROIs (with ROIs coordinate extracted from GLM1 using leave-one-out procedure) of three brain structures in the population of 24 participants. Each group of bars (three groups in each ROI) shows the β coefficients for bundles in partial physically dominating relationships on different ICs: low versus mid, mid versus high, and low versus high. Orange bars represent the higher preference level and blue bars represent the lower preference level. The bars show the mean ± SEM. Download Figure 3-2, TIF file. [file ns-JN-RM-1555-20-s01.tif]

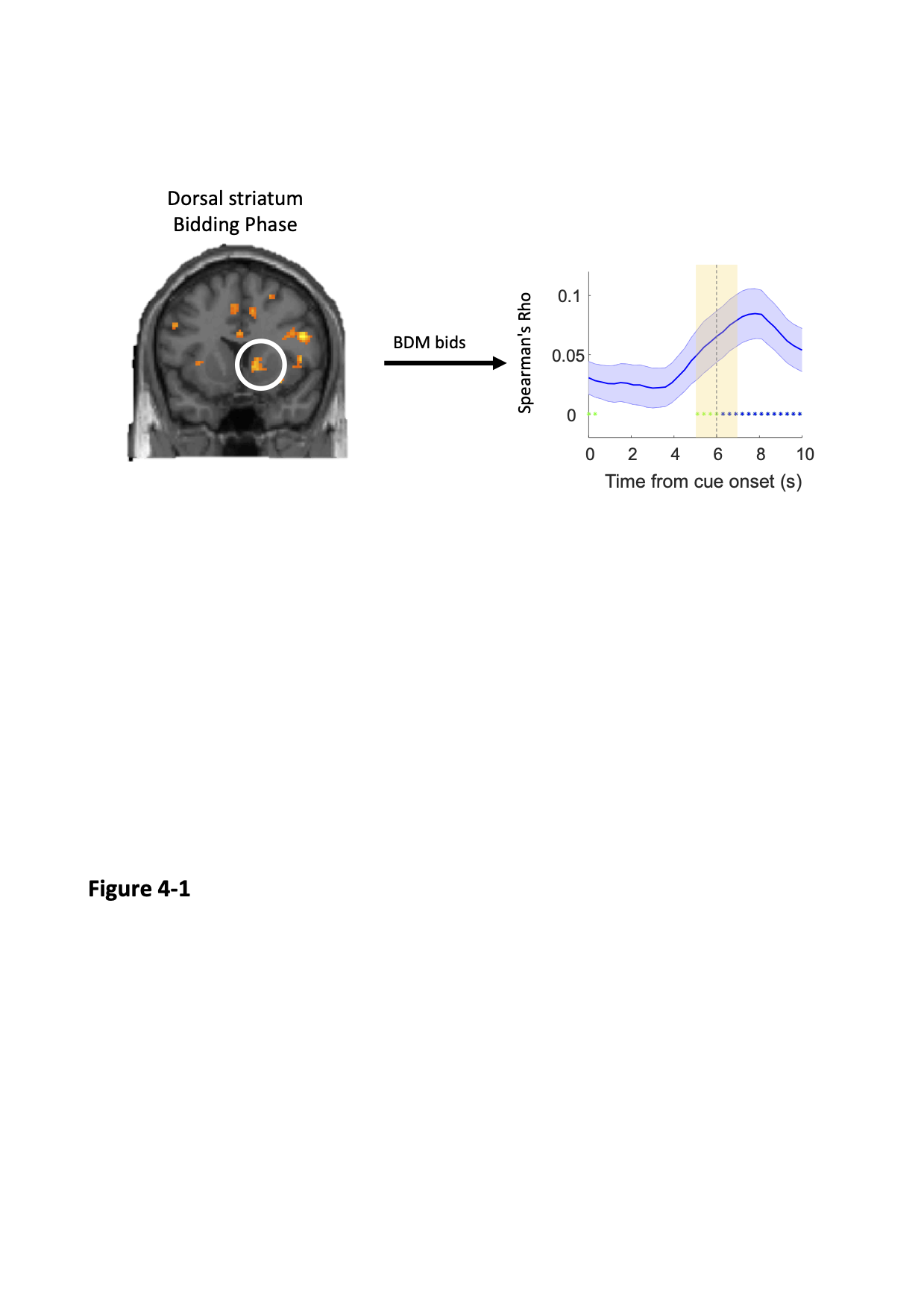

Supplement: Extended Data Figure 4-1 — Dorsal striatum activation during bidding phase (GLM3: activation correlated with the amount of BDM bids; threshold p < 0.005, extent threshold ≥ 10 voxels). Brain map (left) shows dorsal striatum activity during bidding phase. Spearman's rank analysis (right) showed significant Rho coefficient across bids during bidding phase in dorsal striatum. Download Figure 4-1, TIF file. [file ns-JN-RM-1555-20-s03.tif]
